# Marine biodiversity at the end of the world: Cape Horn and Diego Ramírez islands

**DOI:** 10.1371/journal.pone.0189930

**Published:** 2018-01-24

**Authors:** Alan M. Friedlander, Enric Ballesteros, Tom W. Bell, Jonatha Giddens, Brad Henning, Mathias Hüne, Alex Muñoz, Pelayo Salinas-de-León, Enric Sala

**Affiliations:** 1 Pristine Seas, National Geographic Society, Washington DC, United States of America; 2 Fisheries Ecology Research Laboratory, University of Hawai‘i, Honolulu, Hawai‘i, United States of America; 3 Centre d′Estudis Avançats (CEAB-CSIC), Blanes, Spain; 4 Department of Geography, University of California Los Angeles, Los Angeles, California, United States of America; 5 Remote Imaging Team, National Geographic Society, Washington DC, United States of America; 6 Fundación Ictiológica, Santiago, Chile; 7 Charles Darwin Research Station, Puerto Ayora, Galápagos Islands, Ecuador; University of California Santa Cruz, UNITED STATES

## Abstract

The vast and complex coast of the Magellan Region of extreme southern Chile possesses a diversity of habitats including fjords, deep channels, and extensive kelp forests, with a unique mix of temperate and sub-Antarctic species. The Cape Horn and Diego Ramírez archipelagos are the most southerly locations in the Americas, with the southernmost kelp forests, and some of the least explored places on earth. The giant kelp *Macrocystis pyrifera* plays a key role in structuring the ecological communities of the entire region, with the large brown seaweed *Lessonia* spp. forming dense understories. Kelp densities were highest around Cape Horn, followed by Diego Ramírez, and lowest within the fjord region of Francisco Coloane Marine Park (mean canopy densities of 2.51 kg m^-2^, 2.29 kg m^-2^, and 2.14 kg m^-2^, respectively). There were clear differences in marine communities among these sub-regions, with the lowest diversity in the fjords. We observed 18 species of nearshore fishes, with average species richness nearly 50% higher at Diego Ramírez compared with Cape Horn and Francisco Coloane. The number of individual fishes was nearly 10 times higher at Diego Ramírez and 4 times higher at Cape Horn compared with the fjords. Dropcam surveys of mesophotic depths (53–105 m) identified 30 taxa from 25 families, 15 classes, and 7 phyla. While much of these deeper habitats consisted of soft sediment and cobble, in rocky habitats, echinoderms, mollusks, bryozoans, and sponges were common. The southern hagfish (*Myxine australis*) was the most frequently encountered of the deep-sea fishes (50% of deployments), and while the Fueguian sprat (*Sprattus fuegensis*) was the most abundant fish species, its distribution was patchy. The Cape Horn and Diego Ramírez archipelagos represent some of the last intact sub-Antarctic ecosystems remaining and a recently declared large protected area will help ensure the health of this unique region.

## Introduction

Kelp forests are the foundation of many of the shallow rocky coasts of the world’s cold-water marine habitats, providing food and three-dimensional structure for a wide range of species [1–4]. They produce the largest biogenic structures in the ocean, are important in marine carbon cycles, and constitute one of the most diverse and productive ecosystems on the planet [5–8]. Kelp forests are important recruitment and nursery habitat for numerous species and provide a key link between nearshore and deep-water habitats [2,4,9]. The biomass and persistence of these kelp forests are controlled by many biotic and abiotic factors including disturbance from large wave events, seasonal and interannual nutrient inputs, top-down consumer interactions, and anthropogenic degradation of habitat [10–15]. The relative impacts of these forces are often difficult to tease apart since there have been major reductions in kelp forest community biodiversity over the past few centuries, leading to a lack of understanding of what the natural community was like in the past [16–19].

The vast and complex Magellan Region of extreme southern Chile consists of a diversity of habitats including fjords, deep channels, inland seas, glaciers, and extensive kelp forests that are the product of glacial and post-glacial processes [20], which have created remarkably high levels of terrestrial endemism and the largest temperate forests in the Southern Hemisphere [21–23]. The region still contains largely unfragmented ecosystems, low anthropogenic impacts, and very low population density [23].

The Magellan Region contains the archipelago of Tierra del Fuego, with the main island—Isla Grande de Tierra del Fuego—and numerous smaller islands, including the Cape Horn and Diego Ramírez archipelagos [24–25]. This region is the confluence of water masses from three great oceans (Pacific, Atlantic, and Southern oceans), with a mix of species of temperate and sub-Antarctic distributions that creates a unique area of marine endemism with high biodiversity value [26–27]. The region contains critical habitats for marine mammals of global conservation concern (e.g., humpback whales *Megaptera novaeangliae*, southern right whale *Eubalaena australis*) [28–29], and a number of Important Bird and Biodiversity Areas (IBA) [30–31].

The indigenous Yaghan people, noted by Darwin during the voyage of the Beagle [32], were hunters and gatherers who settled the region ~ 10,000 years ago, and represent the world’s southernmost ethnic group [33–35]. Today only 2,200 people live in the Magellan region, most of them in Puerto Williams, which is located on Navarino Island in the Beagle Channel and is the southernmost town in the world [36]. The Patagonian toothfish (*Dissostichus eleginoides*) and southern hake (*Merluccius australis*) fisheries were once important to the economy of the region, but severe overfishing in recent years has greatly reduced the catch of these species [37–38]. The southern king crab (*Lithodes santolla*) and false king crab (*Paralomis granulosa*) fisheries are currently the most important economic activities in the region, but large declines have recently been noted for these species as well [36].

The marine ecosystems of the Magellan Region are diverse with unique biogeography, yet have been poorly studied to date [39]. The region possesses the southernmost kelp forests in the world and therefore has extremely high biodiversity value. The importance of these shallow water habitats as nurseries for commercially valuable resource species and the interconnectivity among deep and shallow habitats is largely unknown. The vast expanses of unfragmented habitats within the region have been recognized for their pristine condition, but efforts to maintain this healthy ecological state are challenged by a variety of human impacts including: overfishing, aquaculture, tourism, transportation, and insufficient management capacity [36,40]. With these factors in mind, we set out to conduct a comprehensive and integrated assessment of the marine ecosystems of the region to: 1) compare the marine communities under different environmental regimes, 2) establish baselines for future comparisons, 3) conduct the first marine assessment of Diego Ramírez, and 4) help inform management of this unique region, including the potential benefits of increased protection.

## Methods

### Ethics statement

Data were collected by all authors in a collaborative effort. Field work and fish collection permits were granted by the Chilean Fisheries Service under a Technical Memorandum (P.INV N° 224/2016 SUBPESCA). This study was carried out in strict accordance with the recommendations of the Canadian Council on Animal Care guidelines on euthanasia of animals used in science. Animal Care and Use was approved by the Charles Darwin Foundation Animal Care and Use Committee under permit number 2017–002. Fish were euthanized using clove oil prior to preservation, and all efforts were made to minimize suffering. Our data are available at Data Dryad: doi:10.5061/dryad.jf36b.

### Site descriptions

**Fig 1:**
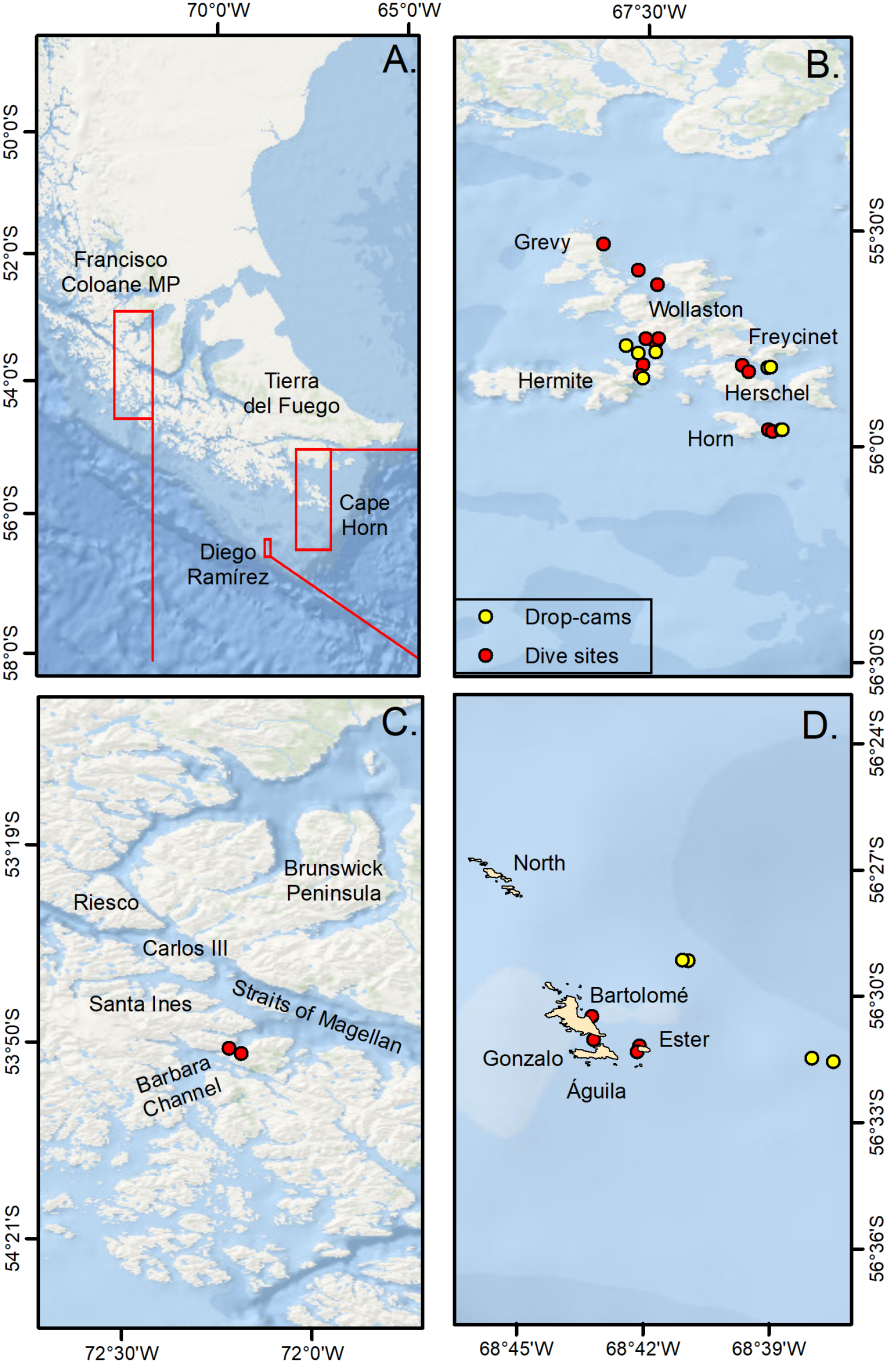
Sampling locations in southern Patagonia. A. Magellan Region, B. Cape Horn Archipelago, C. Francisco Coloane Marine Park, D. Diego Ramírez.

Francisco Coloane Marine Park is located within the western portions of the Straits of Magellan between Santa Inés and Riesco islands and the Brunswick Peninsula, and includes Carlos III Island (Fig 1). The park, established in 2003, was the first marine national park in Chile, and was specifically designated to conserve feeding areas for humpback whales and breeding areas for Magellanic penguins (*Spheniscus magellanicus*) and South American sea lions (*Otaria flavescens*) [41–42]. The 67,197-ha park is also important for other marine mammals such as Antarctic Minke whales (*Balaenoptera bonaerensis*), Orcas (*Orcinus orca*), and southern elephant seals (*Mirounga leonina*).

The waters of the Straits of Magellan are fresher, and cooler than the open shelf waters owing to the effects of melting water from numerous glaciers [43]. The eastward influence of the Pacific by the Antarctic Circumpolar Current (West Wind Drift) reaches Carlos III Island, where a narrow constriction and shallow sill exists, thus constraining water exchange between the Pacific and the Straits [40]. This semi-closed fjord system possesses an extensive and complex seascape that harbors a unique and diverse suite of species [44–45].

Cape Horn is the southernmost headland of the Tierra del Fuego Archipelago, marking the northern boundary of the Drake Passage where three great oceans meet [46]. After European contact in 1616, Cape Horn became a major shipping route for much of the world’s commerce prior to the construction of the Panama Canal [47–49]. The weather around Cape Horn is extreme, owing to intense winds, large waves, strong currents, and icebergs, making it notorious as one of the most hazardous shipping route in the world [50].

Cabo de Hornos National Park encompasses the entire Cape Horn Archipelago and is comprised of a series of islands and islets, including the main landmasses of Wollaston and Hermite islands. It was designated a UNESCO Biosphere Reserve in 2005, and is the world’s southernmost national park [40]. The terrain is almost entirely treeless peat except for some small wooded areas of beech forest (*Nothofagus* spp., [51]). It is one of the world’s hotspot for mosses, liverworts, and lichens, which are resistant to the low temperatures and harsh weather [40,52].

The Diego Ramírez Islands are a small archipelago located on the southern edge of the continental shelf ca. 105 km west-southwest of Cape Horn and ca. 700 km northwest of the South Shetland Islands and the Antarctic Peninsula. The archipelago is divided into a smaller northern group with six islets, and a larger southern group, separated by a 3-km wide pass. The two largest islands, Bartolomé and Gonzalo, both lie in the southern group. Águila Islet, the southernmost land of the group, is at 56°32′9"S. These islands are the southernmost inhabited outpost of South America, and are an important nesting site for numerous seabird species, including the black-browed albatross (*Thalassarche melanophris*), grey-headed albatross (*T*. *chrysostoma*), shy albatross (*Diomedea cauta*), Southern Rockhopper penguin (*Euduptes chrysocome chrysocome*) and Macaroni penguin (*E*. *chrysolophus*) [53–54].

### Benthic surveys

Characterization of the benthos was conducted by scuba divers along two 25-m long transects at each sampling location. Transects were run parallel to the shoreline, with a target depth of 10 m, depending on location of the kelp forest. For sessile and mobile invertebrates, the number of individuals was estimated on 1-m of either side of the transect line (50 m^2^). For colonial organisms (sponges, some cnidarians, bryozoans, and some tunicates) colonies, rather than individuals, were counted. When a species was extremely abundant (i.e. > 500) along the transect, abundance was estimated considering the number of individuals/colonies m^-2^ and scaled to the total area of the transect (50 m^2^). Only non-cryptic invertebrates ≥1 cm were enumerated. A second diver counted the number of kelp (*Macrocystis pyrifera* and *Lessonia* spp.) stipes within 1-m on either side of the transect. Salinity and temperature measures were recorded using a YSI model 556 handheld multiparameter instrument at Francisco Coloane and a RBR concerto multi-channel logger at Cape Horn and Diego Ramírez.

### Giant kelp canopy biomass

Floating canopy of giant kelp was observed over regional scales using the Landsat 8 Operational Land Imager (OLI) multispectral sensor, which provides 30-m spatial resolution imagery over 7 spectral bands in the visible/near-infrared region of the electromagnetic spectrum. Atmospherically-corrected imagery were obtained from the United States Geological Survey (earthexplorer.usgs.gov). Emergent canopy biomass density was estimated from the three sub-regions using Multiple Endmember Spectral Mixing Analysis [55]. This procedure models each pixel as a linear combination of one static kelp canopy endmember and one of 30 seawater endmembers unique to each image. The use of dynamic seawater endmembers accounts for changing water conditions (e.g. phytoplankton blooms, suspended sediments, sunglint) between image dates. The fraction of kelp canopy within each pixel is determined from the model with the lowest root mean squared error. Canopy biomass density was estimated from the derived kelp fraction using an empirical relationship established for giant kelp from diver-based canopy biomass estimates [56]. Regional giant kelp canopy biomass dynamics can be subject to strong seasonal patterns, especially in areas with periodic wave disturbance, nutrient inputs, and increased seasonal light cycles [57–59]. For this reason, only austral summertime imagery was used in the analysis (December 2016 –March 2017) to coincide with anticipated kelp canopy biomass maximums and diver sampling. Areas with persistent cloud cover during this time frame (such as Hermite Island in western Cape Horn) were filled with earlier Landsat 8 imagery for use in figures but were not included in the analysis.

### Fish surveys and collections

At each survey site, a scuba diver counted and sized all fishes within 1-m of either side of a 25 m transect line (50 m^2^). The transect extended to the surface or as far as visibility allowed, including species associated with the kelp canopy and water column. Total fish lengths were estimated to the nearest cm.

Fish collections were conducted opportunistically using several methods. Beach seines (10 x 2 m with 10 mm stretch mesh) were used at Horn and Herschel at Cape Horn and Gonzalo Island at Diego Ramírez. High-density polyethylene traps (87 × 69 × 29 cm) with 4.5 x 1.8 cm openings (http://www.fathomsplus.com) were deployed between 85 and 110 m and baited with ~ 0.5 kg of frozen *Cilus gilberti*. At Diego Ramírez, 3 traps were deployed at each of 2 sites, for 3 hours each, while at Cape Horn, 3 traps were deployed at each of 3 sites, for 3 hours each. Fish were also collected by hand or dip net from beneath stones in the upper to mid-intertidal zone (exposed at low tide).

### Deep Ocean Dropcam surveys

National Geographic’s Deep Ocean Dropcams are high definition cameras (Sony Handycam HDR-XR520V 12 megapixel) encased in a 43-cm diameter borosilicate glass sphere that are rated to 10,000 m depth. We also deployed a Dropcam Mini, encased in a 33-cm diameter borosilicate glass sphere and rated to 5,000 m. This Dropcam Mini housed a Sony Handycam FDR-AX33 4K Ultra-High Definition video with a 20.6 megapixel still image capability. Viewing area per frame for both cameras was between 2–6 m^2^, depending on the steepness of the slope where the Dropcam landed. Cameras were baited with ~ 1 kg of frozen fish and deployed for 6 to 9 hrs.

The relative abundance of each species was calculated as the maximum number of individuals per frame (MaxN). The substrata for each Dropcam deployment was classified into standard geological categories following Tissot et al. [60]: mud (M), sand (S), pebble (P), cobble (C), boulder (B), continuous flat rock (F), diagonal rock ridge (R), and vertical rock-pinnacle top (T). Seafloor type was defined by a two-letter code representing the approximate percent cover of the two most prevalent substrata in a habitat patch. The first character represented the substratum that accounted for at least 50% of the patch, and the second represented the second most prevalent substratum accounting for at least 30% of the patch.

### Statistical analyses

Comparisons of kelp canopy biomass density based on Landsat 8 OLI data among sub-regions (Francisco Coloane, Cape Horn, and Diego Ramírez) was conducted using using a generalized linear model with poisson distribution and log link function. *Post hoc* comparisons between sub-regions were tested using contrasts of the least squares means. *In-situ* measures of kelp taxa densities, and benthic assemblage characteristics (e.g., species richness, numerical abundance, diversity, and eveness) among sub-regions were conducted using a Kruskal-Wallis rank-sum test (*X*^2^), with a Steel-Dwass test for unplanned multiple comparisons in the case of a significant main effect [61]. Benthic taxa diversity was calculated from the Shannon-Weaver diversity index [62]: H´ = -Ʃ p_i_ ln(p_i_), where *p*_*i*_ is the proportion of all individuals counted that were of taxa *i*. The evenness component of diversity was expressed as: J = H´/ln(S), where S is the total number of species present [63]. Benthic taxa were categorized by functional groups based on published literature and were as follows: passive suspension feeder, active suspension feeder, herbivorous/browser, carnivorous, omnivorous, and deposit feeder [64]. Correlations between pooled sea urchin densities and densities of the two kelp genera (*Macrocystis* and *Lessonia*) were compared using Spearman’s rank-order correlation (ρ).

Drivers of benthic and fish assemblage structure were investigated using permutation-based multivariate analysis of variance (PERMANOVA, [65]). A Bray–Curtis similarity matrix was created from abundance of benthic taxa, benthic functional groups, and fish species. Sub-region was treated as a fixed factor. Prior to analysis, benthic taxa and functional group abundance data were ln(x+1) transformed, and fish species abundance was 4th-root-transformed. Interpretation of PERMANOVA results was aided using individual analysis of similarities (ANOSIM), and similarity percentages analysis (SIMPER). The ANOSIM R statistic represents pairs of sub-regions that are either well separated (R > 0.75), overlapping but clearly different (R > 0.5), or barely separable at all (R < 0.25). SIMPER identified the taxa most responsible for the percentage dissimilarities between islands using Bray-Curtis similarity analysis of hierarchical agglomerative group average clustering [66]. Principal Coordinate Analysis (PCO) was used to compare benthic and fish assemblage structure among sub-regions. All PERMANOVA, PCOs, and SIMPER analyses were conducted using Primer v6 [65].

## Results

We conducted a total of 35 transects at 18 locations within the Magellan Region (Francisco Coloane MP = 4, Cape Horn = 23, Diego Ramírez = 8). Transect depths averaged 11.2 ± 2.5 m (range: 7–15 m). During the expedition, water temperatures at 10 m around Francisco Coloane MP averaged 8.8°C (± 0.2), while temperatures were nearly a degree higher at Cape Horn ($\bar{X}=9.7^{\text{o}}\text{C}\;\pm \;0.2$) and Diego Ramírez ($\bar{X}\;=9.7^{\text{o}}\text{C}\;\pm \;0.1$). Salinity averaged 30.1‰ (± 0.1) at Francisco Coloane, 33.1‰ (± 0.2) at Cape Horn, and 33.5‰ (± 0.1) at Diego Ramirez.

### Benthic communities

**Fig 2:**
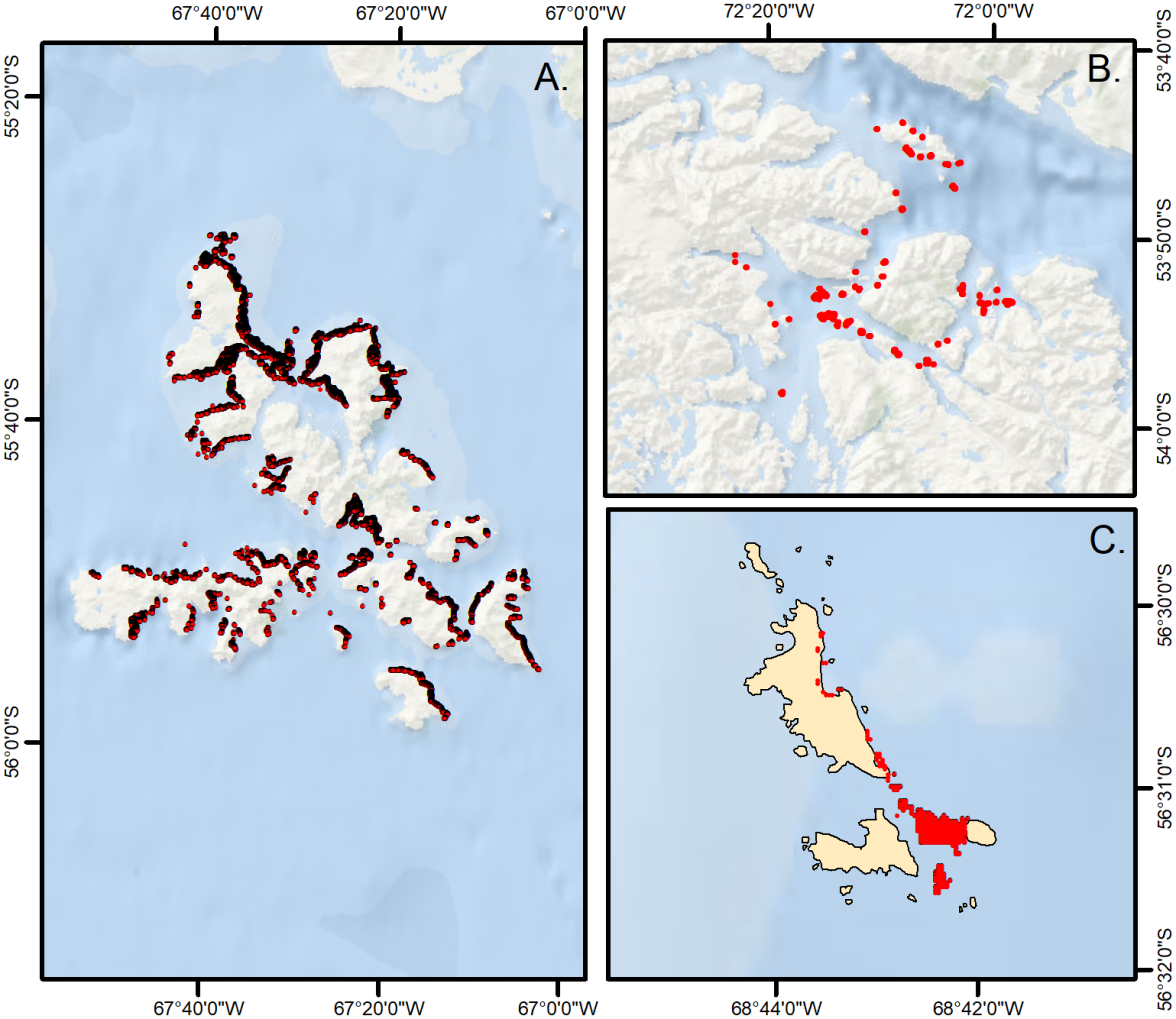
Kelp areal coverage derived from Landsat 8 satellite Operational Land Imager multispectral sensor. A.–Cape Horn, B. Francisco Coloane Marine Park, C. Diego Ramírez.

Kelp forests were the dominant nearshore marine ecosystem in the Magellan Region, with the giant kelp *Macrocystis pyrifera* being the most conspicuous component of this community. In many locations, the large brown seaweed *Lessonia* spp. formed dense understories within the *Macrocystis* canopy. Based on Landsat 8 OLI data, kelp canopy biomass was most dense at the Cape Horn Archipelago with a mean canopy biomass density of 2.51 kg m^-2^ (± 1.27 sd), followed by Diego Ramírez with 2.29 kg m^-2^ (± 0.78 sd), and Francisco Coloane with 2.14 kg m^-2^ (± 1.07 sd). Canopy biomass density was significantly higher at Cape Horn compared to Diego Ramírez, with was in turn significantly higher than Francisco Coloane (*X*^*2*^ = 44.7, p < 0.001; CH > DR > FC). Kelp extent was higher on the eastern and northern coasts of the Cape Horn Archipelago, likely due to shelter from the prevailingly wind and swell that originate from the west (Fig 2).

**Fig 3:**
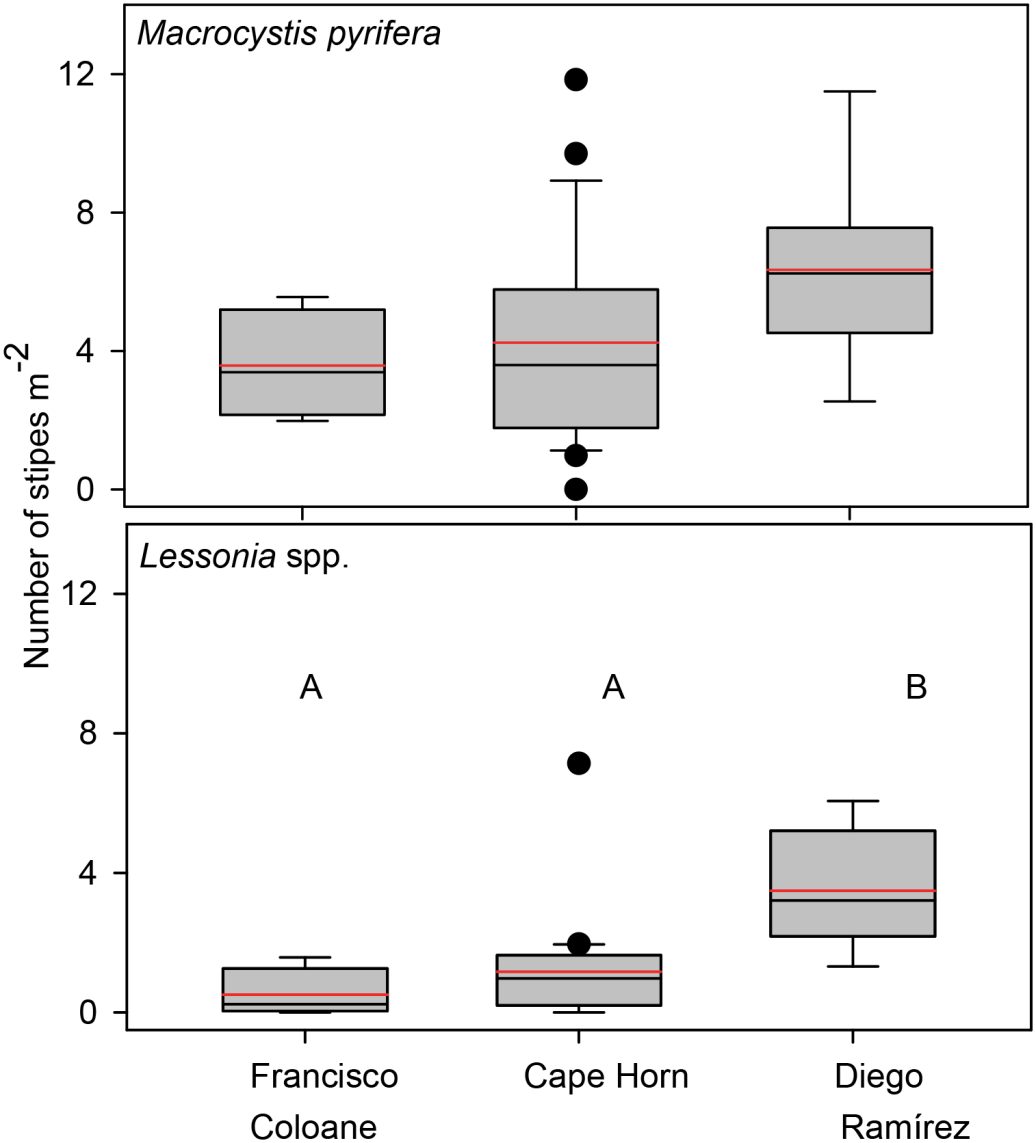
Stipe densities of *Macrocystis pyrifera* and *Lessonia* spp. among the three sub-regions. Box plots showing median (black line), mean (red dashed line), upper and lower quartiles, and 5th and 95th percentiles. Kruskal-Wallis Rank Sum comparisons among regions were statistically different for *Lessonia* spp. (*X*^*2*^ = 13.3, p = 0.001) but not for *Macrocystis pyrifera* (*X*^*2*^ = 4.9, p = 0.08). Regions with the same letter are not significantly different (Steel-Dwass unplanned multiple comparisons procedures, a = 0.05).

Overall *in situ* densities of *M*. *pyrifera* ($\bar{X}\;=4.65\;\pm \;2.86\;\text{no}.\;{\text{m}}^{-2}$) were nearly three times higher than densities of *Lessonia* spp. ($\bar{X}\;=1.62\;\pm \;1.78$, *X*^*2*^ = 24.1, p<0.001). Stipe densities of *M*. *pyrifera* were not significantly different among sub-regions (*X*^*2*^ = 4.9, p = 0.08), although densities at Diego Ramírez were 77% higher than at Francisco Coloane and 50% higher than at Cape Horn (Fig 3). Densities of *Lessonia* spp. were significantly higher at Diego Ramírez compared with Cape Horn and Francisco Coloane (*X*^2^ = 13.3, p = 0.001), which were statistically indistinguishable despite 2-fold high densities at Cape Horn compared to Francisco Coloane.

We recorded 122 invertebrate taxa from 18 classes or infraclasses and 10 phyla during our surveys (S1 Table). Mollusks were the richest phyla with 32 taxa, followed by echinoderms with 20, and sponges with 18. Of the mollusks, gastropods were by far the most specious and abundant. The average number of benthic taxa per transect was highest in the Cape Horn Archipelago and nearly 50% lower at Francisco Coloane (Table 1). The order of magnitude greater abundance of individuals at Cape Horn was driven by the bivalve *Gaimardia trapesina*. If this species is excluded, average numerical density was still significantly greater at Cape Horn ($\bar{X}\;=13.0\;\pm \;9.5$) compared with Diego Ramírez (p = 0.02), but not Francisco Coloane (p = 0.31). Diversity and evenness were both highest at Diego Ramírez and lowest in Francisco Coloane MP.

**Table 1:** Benthic assemblage characteristics among sub-regions. Diversity is Shannon-Wiener H′(log_e_), Evenness is J = H’/ln(S). Statistical results of Kruskal-Wallis rank-sum test (*X*^2^) with Steel-Dwass test for unplanned multiple comparisons. Underlined sub-regions are not significantly different (α = 0.05). Francisco Coloane = FC, Cape Horn = CH, Diego Ramírez = DR.

Metric	Francisco Coloane	Cape Horn	Diego Ramírez	*X*^2^	P	Multiple comparisons
Species (S)	15.00 (5.48)	29.87 (7.61)	25.50 (3.63)	11.08	0.004	CH DR FC
No. m^-2^	7.13 (10.32)	50.22 (82.63)	5.47 (1.05)	9.45	0.009	CH FC DR
Diversity	1.37 (0.67)	1.95 (0.80)	2.50 (0.22)	6.51	0.039	DR CH FC
Evenness	0.51 (0.25)	0.59 (0.23)	0.77 (0.04)	8.34	0.015	DR CH FC

There was a significant difference in the assemblages of benthic taxa among sub-regions (PERMANOVA Pseudo-F_2,34_ = 7.64, p < 0.001). Benthic assemblages based on taxa abundance at Francisco Coloane were distinct from Diego Ramírez (ANOSIM R = 0.987) and Cape Horn (R = 0.898). Although also significant (p<0.001), the benthic assemblages between Cape Horn and Diego Ramírez were more similar to one another compared to the other pair-wise comparisons (R = 0.634).

**Fig 4:**
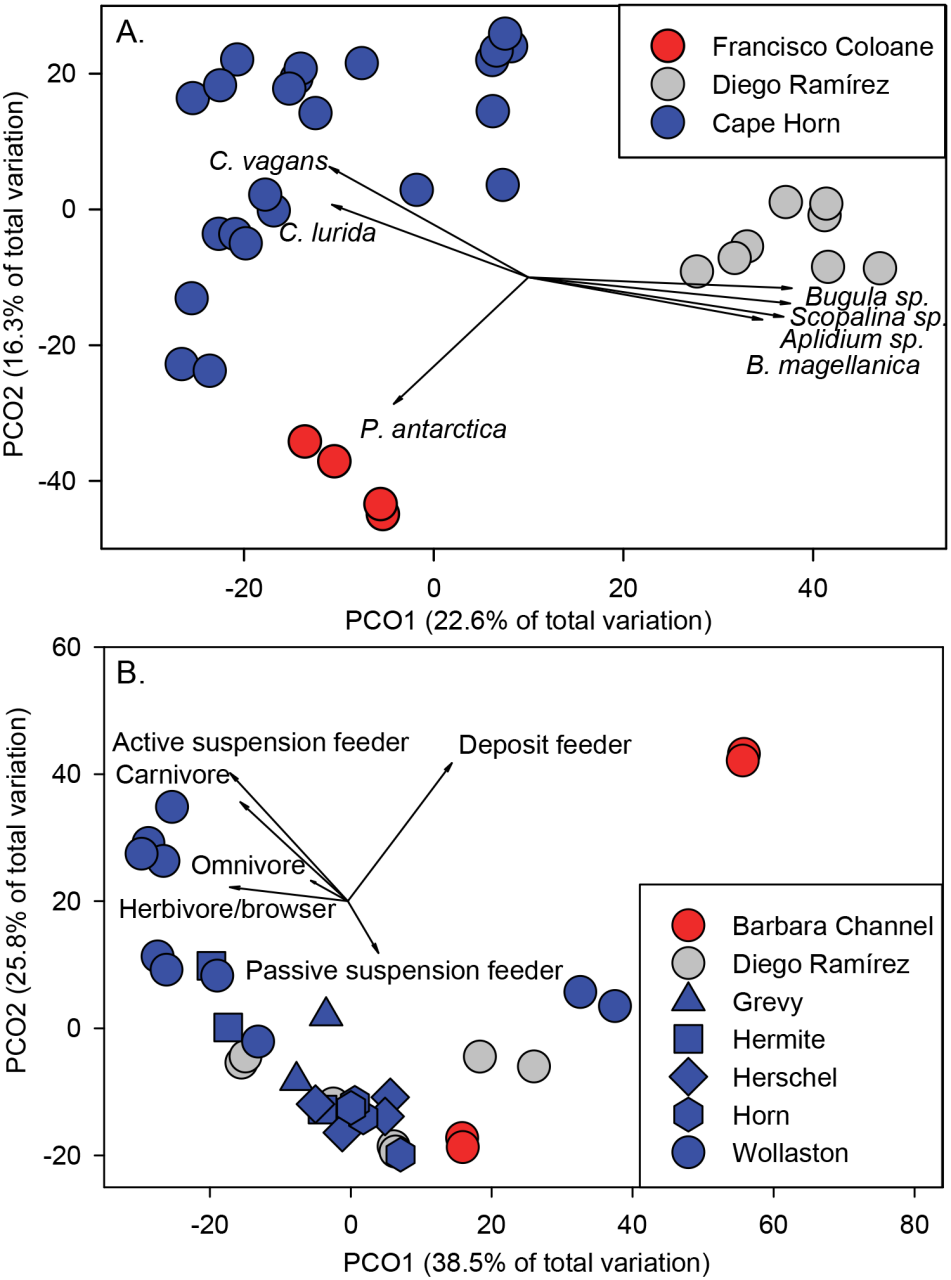
Principle coordinates analysis of A. benthic taxa by sub-region and, B. functional groups by island within sub-regions. Benthic taxa and functional group abundance data were ln(x+1) transformed prior to analyses. Vectors are the primary taxa driving the ordination (Pearson Product movement correlations ≥ 0.6). *C*. *vagans*–*Campylonotus vagans*, *C*. *lurida*–*Cosmasterias lurida*, *P*. *antarctica*–*Porania antarctica*.

There was clear separation of sampling locations by sub-region in ordination space and relatively high concordance within sub-regions based on taxa abundance (Fig 4). PCO1 explained nearly 23% of the variation in benthic taxa distribution among the three sub-regions, with the strongest separation between Diego Ramírez and the other two sub-regions. PCO2 explained an additional 16% of the variation and separated Cape Horn from Francisco Coloane. The sea star *Porania antarctica* drove the separation of Francisco Coloane, while the sea star *Cosmasterias lurida* and the painted shrimp *Campylonotus vagans* drove the separation of sites around Cape Horn. The encrusting red sponge *Scopalina* sp., the colonial tunicate *Aplidium* sp., the colonial arborescent bryozoan *Bugula* sp., and the encrusting bryozoan *Beania magellanica* accounted for the separation of Diego Ramírez.

The sea cucumber *Cladodactyla crocea croceoides* accounted for 73.1% of the total numerical abundance at Francisco Coloane, followed by the colonial tunicate *Didemnum studeri* (7.3%), the barnacle *Notobalanus flosculus* (6.7%), and the sea urchin *Arbacia dufresnii* (4.6%) (Table 2). The bivalve *Gaimardia trapesina* comprised 74.2% of benthic taxa abundance at Cape Horn, followed by the barnacle *Balanus* cf. *laevis* (4.0%), the false king crab *Paralomis granulosa* (2.7%), and the carnivorous top snail *Argobuccinum ranelliforme* (2.4%). Diego Ramírez showed greater diversity among taxa, with the bryozoan *Bugula* sp. (13.9%), the sea urchin *Loxechinus albus* (12.3%), the sea snail *Tegula atra* (11.3%) being the most abundant benthic taxa around Diego Ramírez.

**Table 2:** Similarity of percentages (SIMPER) for benthic taxa most responsible for the percent dissimilarities between sub-regions using Bray-Curtis similarity analysis of hierarchical agglomerative group average clustering. Values are mean (no. m^-2^) with standard deviations in parentheses. Diss. = Average dissimilarity with one standard deviation of the mean in parentheses. A = Cape Horn and Diego Ramírez, B = Cape Horn and Francisco Coloane, and C = Diego Ramírez and Francisco Coloane.

A Dissimilarity = 90.50	Cape Horn	Diego Ramírez	Diss.	% Diss.
*Gaimardia trapesina*	37.24 (76.68)	0.01 (0.02)	21.0 (0.6)	23.2
*Balanus* cf. *laevis*	1.98 (3.26)	-	6.2 (0.5)	6.8
*Loxechinus albus*	0.6 (1.31)	0.67 (0.26)	4.5 (1.3)	5.0
*Bugula sp*.	0.05 (0.12)	0.76 (0.83)	4.1 (0.7)	4.5
*Didemnum studeri*	0.79 (1.99)	0.22 (0.35)	4.1 (0.4)	4.5
*Tegula atra*	0.34 (1.12)	0.62 (0.61)	4.0 (0.9)	4.4
*Pagurus comptus*	0.73 (1.43)	-	3.3 (0.6)	3.7
B Dissimilarity = 95.02	Cape Horn	Francisco Coloane	Diss.	% Diss.
*Gaimardia trapesina*	37.24 (76.68)	-	21.0 (0.6)	22.0
*Cladodactyla crocea croceoides*	-	5.21 (9.87)	14.2 (0.6)	14.9
*Balanus* cf. *laevis*	1.98 (3.26)	-	6.5 (0.5)	6.8
*Didemnum studeri*	0.79 (1.99)	0.52 (0.99)	4.9 (0.5)	5.2
*Pagurus comptus*	0.73 (1.43)	-	3.6 (0.6)	3.8
C Dissimilarity = 95.47	Diego Ramírez	Francisco Coloane	Diss.	% Diss.
*Cladodactyla crocea croceoides*	-	5.21 (9.87)	21.0 (0.7)	21.2
*Bugula* sp.	0.76 (0.83)	-	8.2 (0.9)	8.6
*Loxechinus albus*	0.67 (0.26)	-	7.7 (1.7)	8.0
*Tegula atra*	0.62 (0.61)	-	6.9 (0.9)	7.2
*Notobalanus flosculus*	-	0.48 (0.95)	5.2 (0.6)	5.4
*Scopalina* sp.	0.42 (0.38)	-	5.2 (0.9)	5.4
*Ophiactis asperula*	-	0.33 (0.43)	5.2 (0.9)	5.2

The dissimilarity in benthic assemblages based on taxa abundance between Francisco Coloane and Cape Horn was 95.0% and was driven by the abundance of *G*. *trapesina* at Cape Horn and *C*. *crocea croceoides* in the fjords. The dissimilarity between Francisco Coloane and Diego Ramírez was also high (95.5%), with *Bugula* sp., *L*. *albus*, and *T*. *atra* at Diego Ramírez and *C*. *crocea croceoides* in the fjords driving the difference. The dissimilarity between Cape Horn and Diego Ramírez was 90.5% with *G*. *trapesina* and *B*. *laevis* at Cape Horn and *Bugula* sp., and *L*. *albus* at Diego Ramírez accounting for the differences.

### Benthic functional groups among regions

Active suspension feeders comprised 79.5% of overall numerical abundance within the benthic assemblages, of which *G*. *trapesina* accounted for 87.8% of this total. If this species is excluded, then active suspension feeders accounted for 32.1% of functional group abundance, followed by carnivores (31.7%), herbivores/browsers (22.2%), and passive suspension feeders (10.5%).

There was a significant difference in the assemblages of benthic functional groups among sub-regions (Pseudo-F_2,34_ = 6.37, p < 0.001). Benthic assemblages based on taxa at Francisco Coloane were distinct from Diego Ramírez (ANOSIM R = 0.741) and Cape Horn (R = 0.711), but indistinguishable between Cape Horn and Diego Ramírez (R = 0.091). Benthic assemblage structure based on abundance by functional group showed less separation among sub-regions, but identified a few unique locations that stood out from the rest (Fig 4). The two transects at Station 1 located in Canal Barbara within Francisco Coloane MP were extreme outliers from all other stations and were explained by the high abundance of the deposit feeding ophiuroid, *Ophiactis asperula*. Despite deposit feeders comprising only 1.1% of overall functional group abundance, this species accounted for 59.1% of the abundance at this location. Active suspension feeders (primarily *G*. *trapesina* and *Balanus* sp.), carnivores (primarily the sea star *C*. *lurida*), and the predatory sea snail *Argobuccinum ranelliforme* were most responsible for separating Stations 13 and 14 at Wollaston Island, within the Cape Horn Archipelago, from most other locations.

Sea urchins are important components of the herbivore/browser functional assemblage. Overall densities of sea urchins were highest at Cape Horn followed by Diego Ramírez, with Francisco Coloane having 9 to 12 times lower densities, respectively, compared to the other locations (Table 3). *Loxechinus albus* accounted for 59% of all sea urchins, with similar densities between Cape Horn and Diego Ramírez, but they were completely absent from Francisco Coloane. *Arbacia dufresnii* and *Pseudechinus magellanicus* comprised an additional 21% and 18%, respectively, of overall sea urchin abundance. There were no significant correlations between the total density of all sea urchins combined and densities of either *Lessonia* (ρ = 0.20, p = 0.26) or *Macrocystis* (ρ = 0.23, p = 0.18). The sea star *C*. *lurida* is a predator on sea urchins and densities of this species was an order of magnitude higher at Cape Horn ($\bar{X}\;=\;0.43\;\pm \;0.42$), compared with both Francisco Coloane ($\bar{X}\;=\;0.04\;\pm \;0.03$) and Diego Ramírez ($\bar{X}\;=\;0.02\;\pm \;0.02$, *X*^*2*^ = 16.6, p = 0.003, CH > FC = DR).

**Table 3:** Sea urchin numerical abundances among sub-regions. Values are mean (no. m^-2^) with standard deviations in parentheses.

Sea urchin species	Francisco Coloane	Cape Horn	Diego Ramírez	Percentage of total
*Loxechinus albus*	-	0.60 (1.31)	0.67 (0.26)	59.1
*Arbacia dufresnii*	0.06 (0.06)	0.22 (0.43)	0.17 (0.18)	20.9
*Pseudechinus magellanicus*	0.03 (0.04)	0.35 (0.43)	0.01 (0.02)	18.1
*Austrocidaris canaliculatum*	0.01 (0.01)	0.02 (0.04)	0.01 (0.01)	1.4
Percentage of total	4.7	55.6	39.7	

### Fishes

A total of 18 species of fishes from 12 families were observed during shallow water (<40 m) surveys (Table 4). Of these, 14 were observed on quantitative transects, with the average size of all species combined only 9.7 cm TL (± 4.9). The blennioid *Calliclinus geniguttatus* (n = 2, $\bar{X}\;=26.5\;\pm \;4.9\;\text{cm}\;\text{TL}$) and the southern hagfish *Myxine australis* (n = 3, $\bar{X}\;=\;21.7\;\pm \;7.6\;\text{cm}\;\text{TL}$) were the only two species larger than 20 cm TL observed on transects.

**Table 4:** Shallow water fish species observed during surveys in the Magellan Region. Mean total length (TL) in cm are from quantitative underwater transects unless otherwise noted.

Order	Family	Species	Mean TL (sd)
Myxiniformes	Myxinidae	*Myxine australis*	21.7 (7.6)
Carcharhiniformes	Scyliorhinidae	*Schroederichthys bivius*^+^	35.0
Rajiformes	Arhynchobatidae	*Bathyraja* *magellanica*^+^	25.0
Clupeiformes	Clupeidae	*Sprattus fuegensis*^#^	7.0 (1.0)
Scorpaeniformes	Sebastidae	*Sebastes oculatus*	24.0
	Agonidae	*Agonopsis chiloensis*	9.0 (1.4)
Perciformes	Zoarcidae	Austrolycus depressiceps*	15.0 (10.0)
		*Piedrabuenia ringueleti*	12.0
	Bovichtidae	*Cottoperca trigloides*	11.0 (7.6)
	Nototheniidae	*Paranotothenia magellanica*	10.6 (3.2)
		*Patagonotothen brevicauda*	10.5 (1.2)
		*Patagonotothen cornucola*	10.8 (3.4)
		*Patagonotothen sima*	6.0 (1.8)
		*Patagonotothen squamiceps*	7.4 (2.3)
		*Patagonotothen tessellata*	8.7 (3.1)
	Eleginopsidae	*Eleginops maclovinus*^#^	26.0
	Harpagiferidae	Harpagifer bispinis*	4.5 (0.8)
	Labrisomidae	*Calliclinus geniguttatus*	26.5 (4.9)

*Intertidal hand collection only,

^+^Trap only,

^#^Beach seine only.

Collections in the rocky intertidal yielded four species, two cod icefishes (*Patagonotothen cornucola*, *P*. *sima*), the Magellan plunderfish (*Harpagifer bispinis*) and an eelpout (*Austrolycus depressiceps*), with the latter two species only observed in the rocky intertidal zone. Beach seining yielded one Patagonian blenny or rockcod (*Eleginops maclovinus*, 26 cm TL) at Herschel Island, 40 Fueguian sprat (*Sprattus fuegensis*, $\bar{X}\;=7\;\text{cm}\;\text{TL}$) at Horn Island, and the Magellanic rockcod (*P*. *magellanica*), which were caught at Horn Island (n = 1, 5.0 cm TL), and Gonzalo Island (n = 5, $\bar{X}\;=7.5\;\pm \;1.8\;\text{cm}\;\text{TL}$).

Traps resulted in the capture of one narrowmouthed catshark (*Schroederichthys bivius*, 35 cm TL), one Magellanic ray (*Bathyraja magellanica*, ~ 25 cm disk width), two cod icefish (*P*. *cornucola*, 12 and 14 cm TL), and 24 southern hagfish (range 30–50 cm TL). Trap catch rates were similarly low between Diego Ramírez (0.66 [± 0.63 sd] fish per trap hour, n = 18 total trap hrs) and Cape Horn (0.59 [± 0.43 sd] fish per trap hour, n = 27 total trap hrs).

**Fig 5:**
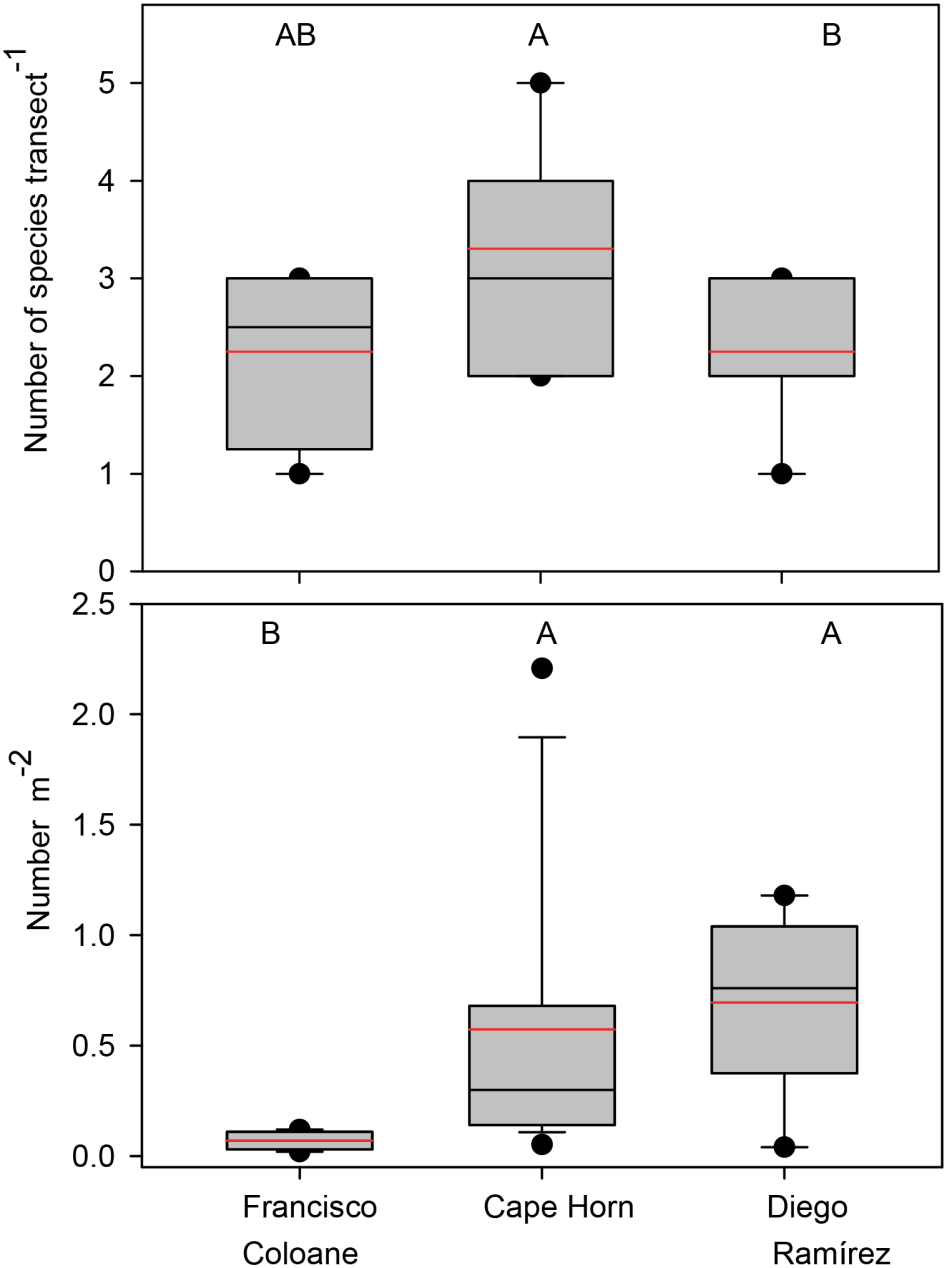
Fish assemblage characteristics among the three sub-regions. Box plots showing median (black line), mean (red dashed line), upper and lower quartiles, and 5th and 95th percentiles. Kruskal-Wallis Rank Sum comparisons among regions were statistically different for species richness (*X*^*2*^ = 13.3, p = 0.001) and numerical abundance (*X*^*2*^ = 4.9, p = 0.08). Regions with the same letter are not significantly different (Steel-Dwass unplanned multiple comparisons procedures, a = 0.05).

The number of fish species observed on transects was significantly higher (X^2^ = 7.1, p = 0.03) at Diego Ramírez ($\bar{X}\;=3.3\;\pm \;1.06$) compared with Cape Horn ($\bar{X}\;=\;2.3\;\pm \;0.7$) and Francisco Coloane ($\bar{X}\;=2.3\;\pm \;1.0$), which were not significantly different from one another. The number of individual fishes was nearly 10 times higher at Diego Ramírez ($\bar{X}\;=0.70\;\pm \;0.38$) compared with Francisco Coloane ($\bar{X}\;=0.07\;\pm \;0.04$), and four times higher at Cape Horn ($\bar{X}\;=0.57\;\pm \;0.63$) compared with Francisco Coloane (Fig 5).

**Fig 6:**
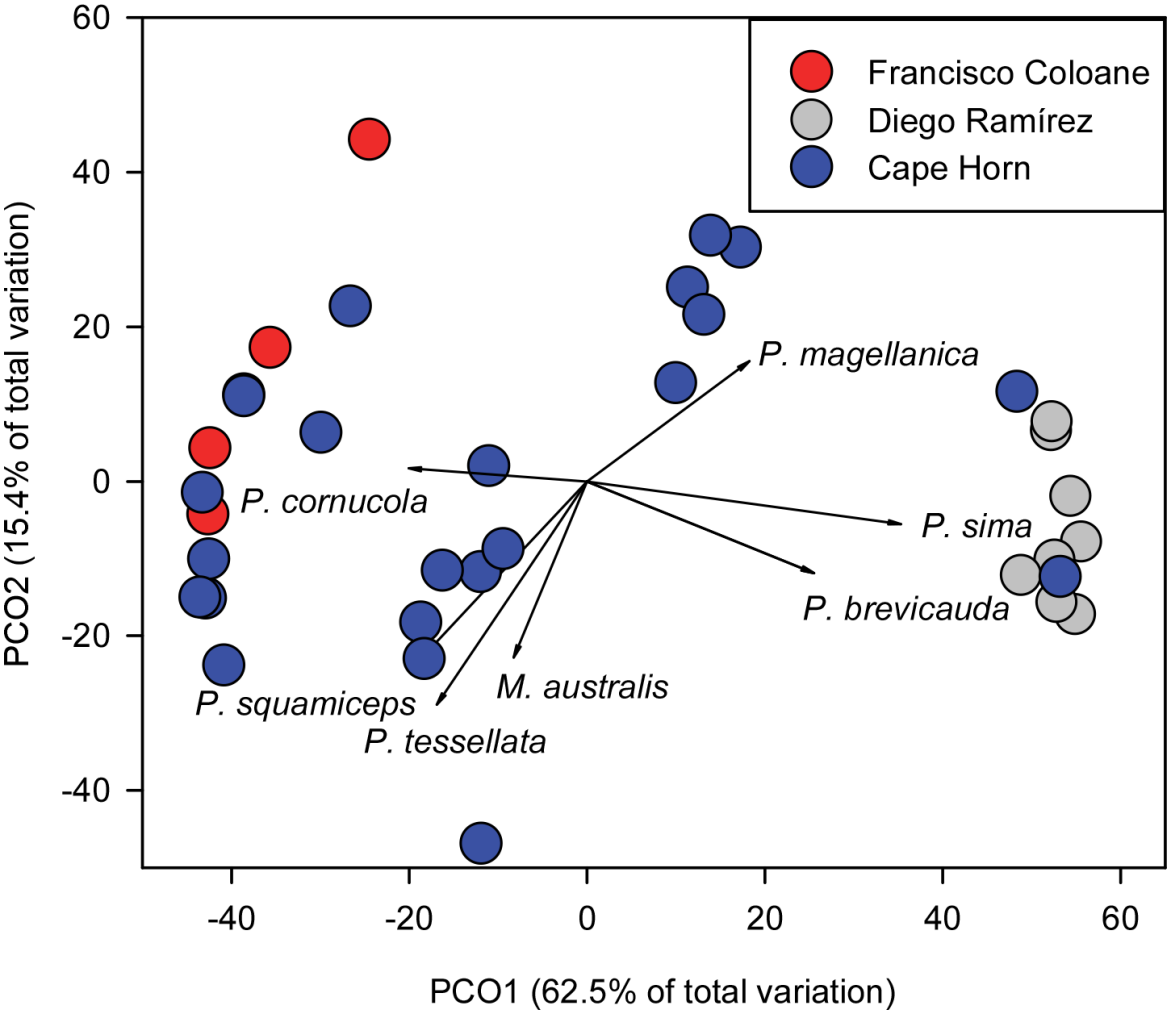
Principle coordinates analysis of fish species numerical abundance by sub-region. Data were 4^th^ root transformed prior to analyses. Vectors are the primary taxa driving the ordination (Pearson Product movement correlations ≥ 0.3). P. spp.–*Patagonotothen* spp. except for *P*. *magellanica*—*Paranotothenia magellanica*. *M*. *australis*—*Myxine australis*, *C*. *geniguttatus—Calliclinus geniguttatus*.

There were clear differences in the fish assemblages between Diego Ramírez and the other two sub-regions, with PCO1 explaining 62.5% of the total variation (Fig 6). These differences were driven by the cod icefishes *P*. *sima* and *P*. *brevicauda* at Diego Ramírez. The two stations at Horn Island, at the extreme southern tip of the Cape Horn Archipelago, were most similar to the fish assemblage at Diego Ramírez, likely due to similar exposed oceanic environments. Another cod icefish, *P*. *cornucola*, was most common in the fjords and protected locations within the Cape Horn Archipelago.

There was 100% dissimilarity in the fish assemblages between Francisco Coloane and Diego Ramírez, which was primarily driven by the high density of *P*. *sima* at Diego Ramírez (Table 5). Similarly, there was a 93% dissimilarity in the fish assemblages between Cape Horn and Diego Ramírez, driven by the high density of *P*. *sima* at Diego Ramírez and *P*. *tessellata* at Cape Horn (Fig 6). Dissimilarly between Cape Horn and the fjords was 79% with low densities of all species in the fjords.

**Table 5:** Similarity of percentages (SIMPER) for fish species most responsible for the percent dissimilarities between sub-regions using Bray-Curtis similarity analysis of hierarchical agglomerative group average clustering. Values are mean (no. m^-2^) with standard deviations in parentheses. Diss. = Average dissimilarity with one standard deviation of the mean in parentheses.

A Dissimilarity = 79.15	Francisco Coloane	Cape Horn	Diss.	% Diss.
*Patagonotothen tessellata*	0.03 (0.03)	0.33 (0.56)	31.3 (1.0)	39.55
*Patagonotothen squamiceps*	0.01 (0.01)	0.12 (0.17)	20.1 (1.0)	25.37
*Patagonotothen sima*	-	0.03 (0.03)	9.7 (0.7)	12.22
*Patagonotothen cornucola*	0.03 (0.01)	0.06 (0.06)	9.4 (1.0)	11.87
*Paranotothenia magellanica*	-	0.02 (0.05)	6.6 (0.5)	8.38
B Dissimilarity = 100.00	Francisco Coloane	Diego Ramírez	Diss.	% Diss.
*Patagonotothen sima*	-	0.65 (0.37)	76.6 (3.3)	76.57
*Patagonotothen cornucola*	0.03 (0.01)	-	7.3 (0.8)	7.26
*Patagonotothen tessellata*	0.03 (0.03)	-	6.1 (0.6)	6.06
*Patagonotothen brevicauda*	<0.01 (0.01)	0.01 (0.01)	4.0 (0.6)	3.96
C Dissimilarity = 92.99	Cape Horn	Diego Ramírez	Diss.	% Diss.
*Patagonotothen sima*	0.03 (0.03)	0.65 (0.37)	51.5 (2.0)	55.41
*Patagonotothen tessellata*	0.33 (0.56)	-	17.9 (0.7)	19.21
*Patagonotothen squamiceps*	0.12 (0.17)	-	10.1 (0.8)	10.82
*Patagonotothen cornucola*	0.06 (0.06)	-	6.4 (0.9)	6.85

### Mesophotic habitats

We conducted 12 Deep Ocean Dropcam deployments ranging in depth from 53 to 105 m ($\bar{X}=\;79\;\pm \;\text{SD}\;16.5$), with recording times ranging from 89 to 364 min ($\bar{X}=\;231\;\pm \;\text{SD}\;98$, Table 6). The most common habitat encountered was sand and cobble, followed by mud and pebbles. Unique habitats included a diagonal rock ridge at 100 m, and a boulder field at 87 m.

**Table 6:** Deep Ocean Dropcam deployment statistics and associated habitats. Habitats: mud (M), sand (S), pebble (P), cobble (C), boulder (B), continuous flat rock (F), diagonal rock ridge (R), and vertical rock-pinnacle top (T). The first letter represents at least 50% cover by that category, and the second, at least 30% cover. Combined, the two-letter code represents ≥ 80% of the benthic cover at a site. Deploy–deployment.

Deploy.	Sub-region	Island	Lat.	Long.	Time (min)	Depth (m)	Benthic (50%)	Benthic (30%)
1	Cape Horn	Horn	-55.961	-67.194	100	53	S	S
2	Cape Horn	Horn	-55.962	-67.192	270	60	S	S
3	Diego Ramírez	Gonzalo	-56.486	-68.682	130	87	B	C
4	Diego Ramírez	Gonzalo	-56.486	-68.684	360	95	P	C
5	Diego Ramírez	Bartolomé	-56.524	-68.633	355	85	S	S
6	Diego Ramírez	Bartolomé	-56.526	-68.625	186	84	S	C
7	Cape Horn	Freycinet	-55.817	-67.226	167	105	S	C
8	Cape Horn	Freycinet	-55.816	-67.218	364	100	F	F
9	Cape Horn	Hermite	-55.842	-67.514	254	76	M	M
10	Cape Horn	Hermite	-55.783	-67.525	89	75	S	S
11	Cape Horn	Wollaston	-55.781	-67.485	251	63	S	P
12	Cape Horn	Wollaston	-55.767	-67.554	253	65	S	C

Thirty taxa from 25 families, 15 classes, and 7 phyla were observed on our Deep Ocean Dropcam deployments (S2 Table). Sponges were observed on 75% of the deployments and covered as much as 30% of the benthos on one occasion at 95 m on pebble and cobble habitat. Bryozoans were common between 87 and 105 m, and covered up to 20% of the substrate on sand and cobble habitat. Five taxa of cnidarians were observed from four different orders, including hydrocoral (*Errina antarctica*, Stylasteridae), hard coral (*Tethocyathus endesa*, Caryophylliidae), octocoral (Primnoidae), and anemones (*Paranthus niveus*, Actinostolidae). Octocorals and hydrocorals were most frequently observed (17% of deployments), with octocorals the most dominant numerically. The hard coral *T*. *endesa* was only observed on one deployment (100 m on continuous flat rock habitat), where it covered 5% of the benthos.

Of the mobile invertebrates, echinoderms were relatively diverse, with seven species from seven different orders observed. The sea star *C*. *lurida* was the most frequently observed, occurring on 25% of the deployments. Another sea star *Cycethra verrucosa* was the most numerically dominant echinoderm, with eight observed at one site on sand and cobble (84 m), averaging 0.7 individuals per deployment. Three different orders of mollusks were observed, including clams (*Nucula pisum*, Nuculidae), octopus *(Robsonella fontaniana*, Octopodidae) and sea snails *(Adelomelon ancilla* and *Odontocymbiola magellanica*, Volutidae).

The southern hagfish was the most frequently encountered of the mesophotic fishes (50% frequency of occurrence), primarily on sand and cobble habitat in depths ranging from 65 to 105 m. The cod icefish (*P*. *cornucola*) were the next most abundant of these fishes (17% frequency of occurrence), and were found in depths ranging from 85 to 105 m on sand and cobble habitat. The Fueguian sprat was the most abundant fish species by number, averaging 5.7 per site, but was only found at three sites. Their maximum number (MaxN = 29) occurred at 105 m depth in sand and cobble habitat. The Patagonian redfish (*Sebastes oculatus*, Sebastidae) was observed between 84 and 87 m on boulder, sand, and cobble habitat (17% frequency of occurrence). Of the cartilaginous fishes (Chondrichthyes), the narrowmouthed catshark, was observed on one occasion at 87 m in boulder and cobble habitat. The Magallan skate (*Bathyraja magellanica*, Arhynchobatidae) was observed on two occasions between 85 m and 87 m on boulder, cobble, and sand habitat.

## Discussion

The extensive kelp forests that dominate the Magellan Region play a key role in structuring the entire ecosystem of the area [67]. The high heterogeneity in the benthic communities that we observed are likely due to the extreme environmental conditions and the highly complex coastline of the region [67]. The southern Patagonian marine community has affinities with the Antarctic region, distinct from the rest of Chile, with the faunal break (~ 55°S) largely due to the strong currents that sweep through the Straights of Magellan [68]. Although the region is less diverse than in the north of the country, it has high biodiversity value due to relatively high endemism [68]. During the voyage of the Beagle, Darwin noted the lush kelp forests of Tierra del Fuego and the high diversity of species found within them [32]. Despite being the most southern kelp forests in the world, few ecological studies of this important ecosystem have been conducted [39], and prior to our expedition, none had been undertaken at Diego Ramírez. While our sampling effort provided limited inferential power in terms of the long-term drivers of marine biodiversity within the region, is serves as a value baseline for future investigation of this poorly studied region.

Kelp stipe density and overall marine diversity were highest at Diego Ramírez compared with Cape Horn and Francisco Coloane MP, while its regional kelp canopy density was equal to that of Cape Horn but presented less spatial variability. Dayton [16] noted that kelp in wave protected areas of the southern Magellanic fjords appeared brittle and unhealthy due to shading by surface kelp blades. In addition, there is a strong salinity gradient within the region, which ranges from 20 to 25 ppt in the fjords of Francisco Coloane MP, between 30 and 33 ppt within the Cape Horn Archipelago, and 33–34 ppt at Diego Ramírez [69]. The differences in wave exposure and salinity likely account for the differences in kelp densities that we observed. The higher benthic assemblage richness at Cape Horn was likely due to diversity of habitats in the archipelago with numerous protected coves and bays, as well as exposed shorelines.

Many of the kelp forests in the northern hemisphere have been dramatically altered due to the removal of top predators and the associated proliferation of grazing herbivores, with impacts in some areas having occurred centuries ago [2,16–17,70–72]. The herbivore-kelp dynamics in southern Chile differs greatly from those in the northern hemisphere [73–75]. The Magellan Region is described as having dense *Macrocystis* forests with few sea urchins [76–77]. Four sea urchin species (*L*. *albus*, *P*. *magellanicus*, *A*. *dufresnii* and *A*. *canaliculata*) in the region are known to feed on kelp; however, these species subsist primarily on drift algae and rarely graze directly on *Macrocystis* [39]. The *Macrocystis* beds in central-southern Chile do not appear to be controlled by sea urchins in exposed sites, and the main herbivores are the gastropod *T*. *atra* in protected sites [78]. We observed *T*. *atra* in abundance in some sheltered locations during our expedition, particularly around Cape Horn and at Diego Ramírez where they are presumably more exposed.

Kelp forests within this region are dynamic in space and time, and regulated by wave action, interspecific competition, and substratum availability [74,76–77]. Nearshore edges of kelp beds are constrained by interspecific competition with *Lessonia*, while the seaward extent is limited by substrate availability [77]. Dayton [73] also noted that *Macrocystis* distribution and abundance was negatively affected by entanglement with drift algae and heavy settlement by bivalve mollusks on kelp fronds.

In several sheltered locations at Cape Horn, we observed kelp plants being sunk by the weight of dense aggregations of the bivalve *G*. *trapesina* that completely covered the kelp fronds and were being preyed upon by the sea star *C*. *lurida*, which is the most conspicuous predator in the kelp forests of the region [75,79]. Similar observations were made in the fjord region in the 1970s by Dayton [73]. This brooding pelecypod is known to have long larval duration and wide dispersal capabilities due to kelp rafting [80], and was numerically abundant around Wollaston Island but absent from our sampling stations in the fjords. Only a few *G*. *trapesina* individuals were observed at Diego Ramírez.

Off the 122 invertebrate taxa recorded on transects during our study, the vast majority (> 80%) of those with documented distributions were restricted to the southeast Atlantic, southeast Pacific, sub-Antarctic, and Antarctic regions. The combination of the unique oceanographic conditions and heterogeneity in the Chilean coast has resulted in high levels of endemism in many invertebrate groups, with several marine invertebrate taxa showing latitudinal biodiversity patterns, some explained by the presence of Antarctic fauna [27]. The Chilean fjord region is diverse in terms of marine invertebrate fauna but also poorly studied [81], and our study is therefore important in better understanding the biogeography of the region and establishing baselines for future studies.

Unlike kelp forests in other regions of the world, the fish assemblages in the Magellan Region are not a conspicuous component of the community [82]. The nearshore fishes of southern Chile form a distinct biogeographic unit that extends towards the Atlantic, including the Falkland Islands [83]. The number of fish species and species composition that we observed was low but similar to that of a two-year study conducted by Moreno and Jara [82] at Puerto Toro on Navarino Island, just south of the Beagle Channel. Richness of littoral fishes on the Chilean coast shows a progressive decrease toward higher latitudes, with a marked decrease of species south of 40° S [68,84]. This observed pattern is likely due to the absence of species of subtropical origin, primarily herbivorous fishes, as well as the lack of time for these species to have colonized, evolved, and adapted to colder temperatures following the last glaciation [85]. Of the 49 species of fishes reported from the Beagle Channel to 150 m, 67% are endemic to the Magellanic Province [86], and our results are consistent with this, where as 83% of the shallow-water fish species that we observed were endemic to the Magellanic Province.

A recent meta-analysis of global marine biodiversity showed that fish abundance declined steeply toward polar latitudes, whereas invertebrate abundance trended in the opposite direction [87]. These authors suggest that temperature-mediated metabolic rate–dependent mechanisms favored fishes in tropical regions, with fish predation and herbivory constraining mobile macroinvertebrate diversity at these lower latitudes. Conversely, invertebrate richness increased with increases in nutrients and a decrease in the abundance of predatory fishes.

The shallow fish fauna in the region was dominated by the suborder Notothenioidei (cod icefish), represented by the families Bovichtidae, Eleginopsidae, Nototheniidae, Harpagiferidae and Channichthyidae [82,84,86]. These fishes play a key role in the ecosystem, occupying most of the available trophic niches [88–89], although dominated by detritivores that fed primarily on amphipods and isopods associated with the kelp [82]. They are also important prey for sea lions, cormorants (*Phalacrocorax atriceps*), and the magellanic penguin [90–92] that frequent the area.

Examination of mesophotic depths (53–105 m) revealed a diverse assemblage of species. On the rocky slopes and boulder habitat, echinoderms, mollusks, bryozoans, and sponges were abundant. The fjord region is a dynamic mixing zone, resulting from deep-water emergence, where typically deep-water organisms can be found in comparably shallow water [81]. The deep and mid-depth species mix, resulted in novel communities in the fjord region. Furthermore, there is an east-west gradient in species composition, where close to the continent, the glacial silt habitat supports a high biomass of scavengers and predators (sharks and rays). In these habitats, we saw that the scavenging southern hagfish was the most abundant fish encountered on our deployments. Because of the barriers to dispersal, and the dynamic environment, the deep Chilean fjords represent a unique mixing zone of species, with high biodiversity value.

Overfishing has severely impacted the stocks of Patagonian toothfish, southern hake, and king crab, which are the major economic driver of the region [36]. These fisheries previously provided a much greater contribution to the local economy and employment, but declining stocks have caused severe economic hardships and social displacements. Continued fishing at these levels will only hasten the collapse of these important resources.

Climate-mediated changes are occurring to kelp forests worldwide due to increases in temperature, explosions of sea urchin populations, and overfishing, which can act synergistically to exacerbate kelp declines [93–94]. Kelps dominate cold-water coastal zones and can become physiologically stressed at high sea temperatures, particularly when nutrient availability is low [95–96]. However, the Humboldt Current is the only boundary current that is not currently showing signs of tropicalization [93], and this region may therefore be less impacted by climate change compared with kelp forests elsewhere around the world.

While the tropicalization of this region is currently a lower priority threat to these ecosystems, human mediated introductions of exotic aquaculture species present a more immediate concern. From the 1970s to 1990s, efforts to introduce exotic salmonids in Chile were focused on ocean ranching with the intent to establishing wild populations of Chinook salmon (*Oncorhynchus tschawytscha*) in Chiloé Island and the Prat River in the Última Esperanza Fjord (Magellan Region) [97–98]. The industry is currently expanding into the Aysén and Magellan regions, and one of the main reasons for this expansion to more isolated and cooler areas was a large-scale outbreak of an infectious salmon anemia virus between 2008 and 2010 around Chiloé Island [99].

Chinook salmon have invaded nearly the entire Patagonia region [99], constituting a major threat to biodiversity to the area [100]. They have been confirmed reports of Chinook spawning in rivers off the Beagle Channel, and the establishment of spawning populations has the potential to severely impact native fishes and invertebrate populations throughout the region [101]. The observed diet in escaped salmonids includes fishes, crustaceans, insects and molluscs [97,100], likely imposing a strong predatory pressure on schooling fishes and increasing resource competition with native fishes [100,102–104]. By some estimates, if current escape rates are not reduced, escaped salmon may exceed 4.4 million individuals per year, consuming up to 6600 t of pelagic prey [98]. In addition, the copious amounts of feces, unconsumed feed, and dead fish greatly increase the nutrient load into fjords and other sheltered areas with poor circulation, resulting in lethal consequences to the benthic communities associated with these salmon net pens [81]. The introduction of antibiotics, pesticides, and other pharmaceuticals are also concerns associated with salmon farms in the region. The expansion of this industry, with a long history of environmental impact in Chile [105–106] represents a threat to the biodiversity and conservation of the entire ecosystem of the Magellan region.

The Humboldt Current ecosystem remains largely unprotected [107–109]. The islands in Cape Horn and Diego Ramírez and the waters surrounding them are at a key moment due to increasing local and global stressors. The Chilean Government has declared a marine park that would include Diego Ramírez to the southern limits of the exclusive economic zone of Chile. This would protect >100,000 km^2^, and not only help conserve kelp forests, but also essential habitat for important populations of sea lions, sea elephants, dolphins, whales, penguins, petrels, albatrosses and other seabirds. The prohibition of fishing in this large area would help recover stocks of southern hake, southern king crab and Patagonian toothfish, which have been severely overfished in the region, as well as reduce by-catch of albatrosses, rays, sharks and other vulnerable species. Krill and sardines are the base of the entire food web of the region, and their protection would increase the health of the entire ecosystem. The Diego Ramírez and Cape Horn archipelagos are likely connected to southern South America via the West Wind Drift [73,110–111], and protecting this connectivity is key to the sustainability the ecosystem. This large protected area is an essential step in conserving the biodiversity and ecosystem functioning of the entire region.

## Supporting information

S1 Table Invertebrate taxa recorded during shallow (< 20 m) scuba surveys. Func. Grp. = Functional feeding groups: 1 –passive suspension feeder, 2—active suspension feeder, 3—herbivorous/browser, 4—carnivore, 5 -omnivore, and 6—deposit feeder.
(DOCX)

S2 Table Taxa observed on deep sea Drop-cams. (DOCX)
