# Supplementary material for: Marine biodiversity at the end of the world: Cape Horn and Diego Ramírez islands
Source: PLoS One. 2018 Jan 24;13(1):e0189930. doi: 10.1371/journal.pone.0189930 (PMC5783361; doi:10.1371/journal.pone.0189930)
Supplement: S1 Table — Func. Grp. = Functional feeding groups: 1 –passive suspension feeder, 2—active suspension feeder, 3—herbivorous/browser, 4—carnivore, 5 -omnivore, and 6—deposit feeder. (DOCX) [file pone.0189930.s001.docx]

S1 Table. Invertebrate taxa recorded during shallow (< 20 m) scuba surveys. Func. Grp. = Functional feeding groups: 1 – passive suspension feeder, 2 - active suspension feeder, 3 - herbivorous/browser, 4 - carnivore, 5 -omnivore, and 6 - deposit feeder.

| Phylum | Class to Infraclass | Order to Family | Func.  Grp | Taxa |
| --- | --- | --- | --- | --- |
| Annelida | Polychaeta | Serpulidae | 2 | *Apomatus* sp. |
| Annelida | Polychaeta | Chaetopteridae | 2 | *Chaetopterus variopedatus* |
| Arthropoda | Hexanauplia | Balanidae | 2 | *Arossia henryae* |
| Arthropoda | Hexanauplia | Balanidae | 2 | *Austromegabalanus psittacus* |
| Arthropoda | Hexanauplia | Balanidae | 2 | *Balanus* cf. *laevis* |
| Arthropoda | Hexanauplia | Archaeobalanidae | 2 | *Notobalanus flosculus* |
| Arthropoda | Malacostraca | Munididae | 5 | *Munida gregaria* |
| Arthropoda | Malacrostaca | Campylonotidae | 4 | *Campylonotus vagans* |
| Arthropoda | Malacrostaca | Inachidae | 4 | *Eurypodius latreillii* |
| Arthropoda | Malacrostaca | Hymenosomatidae | 4 | *Halicarcinus planatus* |
| Arthropoda | Malacrostaca | Lithodidae | 4 | *Lithodes santolla* |
| Arthropoda | Malacrostaca | Hippolytidae | 4 | *Nauticaris magellanica* |
| Arthropoda | Malacrostaca | Paguridae | 4 | *Pagurus comptus* |
| Arthropoda | Malacrostaca | Lithodidae | 4 | *Paralomis granulosa* |
| Arthropoda | Malacrostaca | Trichopeltariidae | 4 | *Peltarion spinulosum* |
| Arthropoda | Pycnogonida | Pycnogonidae | 4 | Pycnogonidae |
| Brachiopoda | Rhynchonellata | Terebratellidae | 2 | *Magellania venosa* |
| Bryozoa | Gymnolaemata | Beaniidae | 2 | *Beania magellanica* |
| Bryozoa | Gymnolaemata | Bugulidae | 2 | *Bugula* sp. |
| Bryozoa | Gymnolaemata | Cellariidae | 2 | *Cellaria malvinensis* |
| Bryozoa | Gymnolaemata | Microporellidae | 2 | *Microporella hyadesi* |
| Bryozoa | Gymnolaemata | Bitectiporidae | 2 | *Schizomavella* sp. |
| Bryozoa | Stenolaemata | Crisiidae | 2 | *Crisia* sp. |
| Bryozoa | Stenolaemata | Entalophoridae | 2 | *Entalophora* sp. |
| Bryozoa |  |  | 2 | Unid. encrusting small bryozoa |
| Bryozoa |  |  | 2 | Unid. encrusting thin bryozoan |
| Bryozoa |  |  | 2 | Unid. *Schizobrachiella-*like |
| Chordata | Ascidiacea | Polyclinidae | 2 | *Aplidium fuegiense* |
| Chordata | Ascidiacea | Polyclinidae | 2 | *Aplidium magellanicum* |
| Chordata | Ascidiacea | Polyclinidae | 2 | *Aplidium* sp. 1 |
| Chordata | Ascidiacea | Polyclinidae | 2 | *Aplidium* sp. 2 |

S1 Table. Continued.

| Phylum | Class to Infraclass | Order to Family | Func.  Grp | Taxa |
| --- | --- | --- | --- | --- |
| Chordata | Ascidiacea | Styelidae | 2 | *Cnemidocarpa sp.* |
| Chordata | Ascidiacea | Styelidae | 2 | *Cnemidocarpa verrucosa* |
| Chordata | Ascidiacea | Corellidae | 2 | *Corella eumyota* |
| Chordata | Ascidiacea | Didemnidae | 2 | *Didemnum studeri* |
| Chordata | Ascidiacea | Styelidae | 2 | *Polyzoa opuntia* |
| Chordata | Ascidiacea | Pyuridae | 2 | *Pyura chilensis* |
| Chordata | Ascidiacea | Pyuridae | 2 | *Pyura legumen* |
| Chordata | Ascidiacea | Holozoidae | 2 | *Sycozoa gaimardi* |
| Cnidaria | Anthozoa | Isophelliidae | 1 | *Acontiaria* sp. |
| Cnidaria | Anthozoa | Actiniaria | 1 | Actiniaria |
| Cnidaria | Anthozoa | Actinostolidae | 1 | *Actinostola chilensis* |
| Cnidaria | Anthozoa | Alcyoniidae | 1 | *Alcyonium* cf. yepayek |
| Cnidaria | Anthozoa | Actinostolidae | 1 | *Antholoba achates* |
| Cnidaria | Anthozoa | Sagartiidae | 1 | *Anthothoe* sp. |
| Cnidaria | Anthozoa | Sagartiidae | 1 | *Anthothoe chilensis* |
| Cnidaria | Anthozoa | Actiniidae | 1 | *Boloceropsis* sp. |
| Cnidaria | Anthozoa | Actiniidae | 1 | *Bunodactis octoradiata* |
| Cnidaria | Anthozoa | Clavulariidae | 1 | *Incrustatus comauensis* |
| Cnidaria | Anthozoa | Sagartiidae | 1 | *Actinothoe lobata* |
| Cnidaria | Hydrozoa | Campanulariidae | 1 | *Obelia geniculata* |
| Cnidaria | Hydrozoa | Symplectoscyphidae | 1 | *Symplectoscyphus subdichotomous* |
| Echinodermata | Asteroidea | Asteriidae | 4 | *Anasterias antarctica* |
| Echinodermata | Asteroidea | Stichasteridae | 4 | *Cosmasterias lurida* |
| Echinodermata | Asteroidea | Ganeriidae | 4 | *Cycethra verrucosa* |
| Echinodermata | Asteroidea | Echinasteridae | 4 | *Henricia obesa* |
| Echinodermata | Asteroidea | Echinasteridae | 4 | *Henricia studeri* |
| Echinodermata | Asteroidea | Heliasteridae | 4 | *Labidiaster radiosus* |
| Echinodermata | Asteroidea | Odontasteridae | 4 | *Odontaster penicillatus* |
| Echinodermata | Asteroidea | Asterinidae | 4 | *Asterina fimbriata* |
| Echinodermata | Asteroidea | Poraniidae | 4 | *Glabraster antarctica* |
| Echinodermata | Asteroidea | Poraniidae | 4 | *Poraniopsis echinaster* |
| Echinodermata | Asteroidea | Stichasteridae | 4 | *Stichaster striatus* |
| Echinodermata | Echinoidea | Parechinidae | 3 | *Loxechinus albus* |
| Echinodermata | Echinoidea | Arbaciidae | 3 | *Arbacia dufresnii* |

S1 Table. Continued.

| Phylum | Class to Infraclass | Order to Family | Func.  Grp | Taxa |
| --- | --- | --- | --- | --- |
| Echinodermata | Echinoidea | Cidaridae | 4 | *Austrocidaris canaliculata* |
| Echinodermata | Echinoidea | Temnopleuridae | 3 | *Pseudechinus magellanicus* |
| Echinodermata | Holothuroidea | Cucumariidae | 1 | *Cladodactyla crocea croceoides* |
| Echinodermata | Holothuroidea | Cucumariidae | 1 | *Pseudocnus dubius meloninus* |
| Echinodermata | Ophiuroidea | Ophiactidae | 6 | *Ophiactis asperula* |
| Echinodermata | Ophiuroidea | Ophiomyxidae | 6 | *Ophiomyxa vivipara* |
| Echinodermata | Ophiuroidea | Ophiuridae | 6 | *Ophiura (Ophiuroglypha) lymani* |
| Mollusca | Bivalvia | Gaimardiidae | 2 | *Gaimardia trapesina* |
| Mollusca | Bivalvia | Pectinidae | 2 | *Zygochlamys patagonica* |
| Mollusca | Gastropoda | Volutidae | 4 | *Adelomelon ancilla* |
| Mollusca | Gastropoda | Dorididae | 4 | *Doris fontainii* |
| Mollusca | Gastropoda | Ranellidae | 4 | *Argobuccinum pustulosum* |
| Mollusca | Gastropoda | Muricidae | 4 | *Concholepas concholepas* |
| Mollusca | Gastropoda | Discodorididae | 4 | *Diaulula hispida* |
| Mollusca | Gastropoda | Discodorididae | 4 | *Diaulula punctuolata* |
| Mollusca | Gastropoda | Fissurellidae | 3 | *Fissurella pict/oriens* |
| Mollusca | Gastropoda | Fissurellidae | 3 | *Fissurellidea patagonica* |
| Mollusca | Gastropoda | Ranellidae | 4 | Fusitriton magellanicus |
| Mollusca | Gastropoda | Calliostomatidae | 3 | *Margarella violacea* |
| Mollusca | Gastropoda | Nacellidae | 3 | *Nacella deaurata*/*magellanica* |
| Mollusca | Gastropoda | Nacellidae | 3 | *Nacella mytilina* |
| Mollusca | Gastropoda | Naticidae | 6 | *Naticarius* sp. |
| Mollusca | Gastropoda | Buccinidae | 4 | Pareuthria fuscata |
| Mollusca | Gastropoda | Tegulidae | 3 | *Tegula atra* |
| Mollusca | Gastropoda | Polyceridae | 4 | *Thecacera darwini* |
| Mollusca | Gastropoda | Tritoniidae | 4 | *Tritonia challengeriana* |
| Mollusca | Gastropoda | Muricidae | 4 | *Trophon geversianus* |
| Mollusca | Gastropoda | Muricidae | 4 | *Trophon plicatus* |
| Mollusca | Gastropoda | Chromodorididae | 4 | Tyrinna delicata |

S1 Table. Continued.

| Phylum | Class to Infraclass | Order to Family | Func.  Grp | Taxa |
| --- | --- | --- | --- | --- |
| Mollusca | Gastropoda |  | 4 | Unidentified opistobranchia |
| Mollusca | Gastropoda | Muricidae | 4 | *Xymenopsis muriciformis* |
| Mollusca | Polyplacophora | Callochitonidae | 3 | *Callochiton puniceus* |
| Mollusca | Polyplacophora | Leptochitonidae | 3 | *Leptochiton* cf. *medinae* |
| Mollusca | Polyplacophora | Chitonidae | 3 | *Tonicia atrata* |
| Mollusca | Polyplacophora | Chitonidae | 3 | *Tonicia chilensis* |
| Mollusca | Polyplacophora | Chitonidae | 3 | *Tonicia smithii* |
| Mollusca | Polyplacophora | Chitonidae | 3 | *Chiton boweni* |
| Mollusca | Polyplacophora | Mopaliidae | 3 | *Plaxiphora aurata* |
| Nemertea | Anopla | Valenciniidae | 4 | *Baseodiscus aureus* |
| Porifera | Calcarea | Sycettidae | 2 | *Sycon* spp. |
| Porifera | Calcarea | Clathrinidae | 2 | *Clathrina* cf. *fjordica* |
| Porifera | Calcarea | Leucaltidae | 2 | *Leucettusa nuda* |
| Porifera | Demospongiae | Niphatidae | 2 | *Amphimedon maresi* |
| Porifera | Demospongiae | Clionaidae | 2 | *Cliona chilensis* |
| Porifera | Demospongiae | Dysideidae | 2 | *Dysidea* sp. |
| Porifera | Demospongiae | Chalinidae | 2 | *Haliclona cf. porcelana* |
| Porifera | Demospongiae | Hymedesmiidae | 2 | *Hemimycale sp.* |
| Porifera | Demospongiae | Mycalidae | 2 | Mycale (Aegogropila) magellanica |
| Porifera | Demospongiae | Hymedesmiidae | 2 | *Phorbas* sp. |
| Porifera | Demospongiae | Polymastiidae | 2 | *Polymastia* sp. |
| Porifera | Demospongiae | Tethyidae | 2 | *Tethya papillosa* |
| Porifera | Demospongiae | ? | 2 | Unid. yellow sponge |
| Porifera | Demospongiae | Chondrosiidae | 2 | Unid. *Chondrosia*-like |
| Porifera | Demospongiae | ? | 2 | Unid. encrusting black sponge |
| Porifera | Demospongiae | Scopalinidae | 2 | *Scopalina* sp. |
| Porifera | Demospongiae | Mycalidae | 2 | *Mycale* sp. |
| Porifera |  |  | 2 | Unid. grey sponge |
