# Supplementary material for: Marine biodiversity at the end of the world: Cape Horn and Diego Ramírez islands
Source: PLoS One. 2018 Jan 24;13(1):e0189930. doi: 10.1371/journal.pone.0189930 (PMC5783361; doi:10.1371/journal.pone.0189930)
Supplement: S2 Table — (DOCX) [file pone.0189930.s002.docx]

S2 Table. Taxa observed on deep sea Drop-cams.

| **Phylum** | **Class** | **Order** | **Family** | ***Taxa*** | **Common name** | **Freq (%)** | **MaxN** | **Max** |
| --- | --- | --- | --- | --- | --- | --- | --- | --- |
| Porifera |  |  |  |  | Sponge | 75 |  | 0.3 |
| Bryozoa | Gymnolaemata | Cheilostomatida | Microporellidae | *Microporella hyadesi* | Bryozoan | 8.3 | 0.2 |  |
|  |  |  |  |  | Bryozoan | 16.7 | 0.4 |  |
|  |  |  |  |  | Bryozoan |  |  | 0.1 |
|  | Stenolaemata | Cyclostomatida | Entalophoridae | *Entalophora sp.* | Bryozoan | 16.7 | 1.7 | 0.2 |
| Cnidaria | Anthozoa | Actiniaria | Actinostolidae | *Paranthus niveus* | Anemone | 8.3 | 0.4 |  |
|  |  | Alcyonacea | Primnoidae |  | Octocoral | 16.7 | 3.9 |  |
|  |  | Scleractinia | Caryophylliidae | *Tethocyathus endesa* | Coral | 8.3 |  | 0.05 |
|  | Hydrozoa | Anthoathecata | Stylasteridae | *Errina antarctica* | Hydrocoral | 8.3 | 0.2 |  |
|  |  |  |  |  | Hydrocoral | 16.7 | 1 |  |
| Arthropoda | Malacostraca | Amphipoda |  |  | Amphipod | 8.3 | 5 |  |
| Mollusca | Bivalvia | Nuculida | Nuculidae | *Nucula pisum* | Clam | 8.3 | 0.4 |  |
|  | Cephalopoda | Octopoda | Octopodidae | *Robsonella fontaniana* | Octopus | 8.3 | 0.1 |  |
|  | Gastropoda | Neogastropoda | Volutidae | *Adelomelon ancilla* | Sea snail | 8.3 | 0.1 |  |
|  |  |  |  | *Odontocymbiola magellanica* | Sea snail | 8.3 | 0.1 |  |
| Echinodermata | Asteroidea | Forcipulatida | Stichasteridae | *Cosmasterias lurida* | Sea star | 25 | 0.3 |  |
|  |  | Spinulosida | Echinasteridae | *Henricia obesa* | Sea star | 8.3 | 0.3 |  |
|  |  | Valvatida | Ganeriidae | *Cycethra verrucosa* | Sea star | 8.3 | 0.7 |  |
|  |  | Velatida | Pterasteridae | *Pteraster gibber* | Slime star | 8.3 | 0.3 |  |
|  | Echinoidea | Arbacioida | Arbaciidae | *Arbacia dufresnii* | Sea urchin | 8.3 | 0.3 |  |
|  |  | Cidaroida | Cidaridae | *Austrocidaris canaliculata* | Sea urchin | 8.3 | 0.1 |  |
|  | Ophiuroidea | Phrynophiurida | Gorgonocephalidae | *Gorgonocephalus chilensis* | Basket star | 8.3 | 0.1 |  |
| Chordata | Actinopterygii | Clupeiformes | Clupeidae | *Sprattus fuegensis* | Fueguian sprat | 25 | 5.7 |  |
|  |  | Perciformes | Bovichtidae | *Cottoperca trigloides* | Thornfish | 16.7 | 0.3 |  |
|  |  |  | Nototheniidae | *Patagonotothen cornucola* | Cod icefishes | 41.7 | 1 |  |
|  |  | Scorpaeniformes | Sebastidae | *Sebastes oculatus* | Patagonian redfish | 16.7 | 0.2 |  |
|  | Chondrichthyes | Carcharhiniformes | Scyliorhinidae | *Schroederichthys bivius* | Narrowmouth Catshark | 8.3 | 0.1 |  |
|  |  | Rajiformes | Arhynchobatidae | *Bathyraja magellanica* | Magellan skate | 16.7 | 0.2 |  |
|  | Myxini | Myxiniformes | Myxinidae | *Myxine australis* | Southern hagfish | 50 | 1.2 |  |
